# Relation Between the Adsorbed Quantity and the Immersion Enthalpy in Catechol Aqueous Solutions on Activated Carbons

**DOI:** 10.3390/ijms13010044

**Published:** 2011-12-22

**Authors:** Juan Carlos Moreno-Piraján, Diego Blanco, Liliana Giraldo

**Affiliations:** 1 Group of Investigation in Solid Porous and Calorimetry, Department of Chemistry, University of the Andes, Carrera 1 No 18 A 10, Bogotá, Colombia; 2 Department of Chemistry, Faculty of Sciences, University National of Colombia, Carrera 30 No 45-03, Bogotá, Colombia; E-Mails: dablancom@hotmail.com (D.B.); lgiraldogu@bt.unal.edu.co (L.G.)

**Keywords:** activated Carbon, catechol, oxidation, reduction, adsorption, immersion enthalpies

## Abstract

An activated carbon, Carbochem^TM^—PS230, was modified by chemical and thermal treatment in flow of H_2_, in order to evaluate the influence of the activated carbon chemical characteristics in the adsorption of the catechol. The catechol adsorption in aqueous solution was studied along with the effect of the pH solution in the adsorption process of modified activated carbons and the variation of immersion enthalpy of activated carbons in the aqueous solutions of catechol. The interaction solid-solution is characterized by adsorption isotherms analysis, at 298 K and pH 7, 9 and 11 in order to evaluate the adsorption value above and below that of the catechol pK_a_. The adsorption capacity of carbons increases when the solution pH decreases. The retained amount increases slightly in the reduced carbon to maximum adsorption pH and diminishes in the oxidized carbon. Similar conclusions are obtained from the immersion enthalpies, whose values increase with the solute quantity retained. In granular activated carbon (CAG), the immersion enthalpies obtained are between 21.5 and 45.7 J·g^−1^ for catechol aqueous solutions in a range of 20 at 1500 mg·L^−1^.

## 1. Introduction

Catechol, or ortho-hidroxifenol, is used as an antiseptic topic, in photography, in leather dyeing, in tanneries as inhibitor of polymerization, as chemical intermediary, and in many industries as antioxidant. It is also used in laboratories for the detection and determination of many ions.

Catechol frequently contaminates waste water generated by several industries such as: chemistry, rubbers, dyes, photography, pharmaceutics, cosmetics and oil industry. Catechol is more toxic than phenol since it provokes changes in the eritocite function at doses as low as 50 μg·L^−1^ compared to 250 μg·L^−1^ for phenol [1–4].

Phenolic compound adsorption is probably one of the applications most studied in liquid phase for carbon materials. Because of the adsorption capacity, the compounds earlier mentioned from aqueous solution in activated carbons depend on various factors, of which some depend on the solid nature as the porous network and the chemical surface and others on the solution as the pH effect, the dissolved solute quantity, the presence of other possible adsorbates, besides the ionic force and the morphology of the same [5–9].

The determinations of the immersion enthalpy of the activated carbon in different solutions, provide a direct measure of the energy involved in the process, which is not only related to the surface area available to the liquid, but also to the specific interaction between the solid surface and the immersion liquid, allowing evaluation of the value of the enthalpy as a thermodynamic property that characterizes the interaction solid-liquid [10–14].

As the adsorption capacity is modified by the pH solution, producing variation in the solute species for being weak acids, the following is expected for the used activated carbons: one original, one oxidized and one reduced; the isotherms and the immersion enthalpies allow characterizing the influence of the pH in the adsorption capacity. At the same time the content of surface groups modifies the electrochemical behavior of the surface and the interactions with the organic electrolyte that will newly be observable with the proposed determinations.

Hence, this work seeks to identify the effects of some variables involved in the catechol adsorption process in aqueous solution on activated carbon, between the pH effect and the influence of the reduction and oxidation on the activated carbon surface on the adsorption capacity and a study of the immersion enthalpy variation of the activated carbon [1–5,10–17].

## 2. Results and Discussion

### 2.1. Activated Carbon Properties

The physical and chemical characteristics of the activated carbons studied in this work are shown in Tables 1 and 2. Table 1 shows the values for the micropore and mesopore volume. The micropore volume was calculated after applying the Dubinin–Radushkevich (DR) equation to the N_2_ adsorption isotherm, total pore volume was obtained from the amount adsorbed at P/Po 0.99 and the mesopore volume was determined as the difference between these two values.

The results show that the oxidation and reduction treatments done in the activated carbon do not produce significant changes in the textural characteristics of carbonaceous materials, except in terms of the electrochemical properties of the surface.

The reduction treatment of the activated carbon surface with H_2_ was slight given the temperature of 573 K that was used for the reaction; the intention of this is that the change in the surface group content is small and to check if an increase in the catechol adsorption capacity occurs. Small modification is observed in the surface groups and the pH_pzc_, which did not change significantly with regard to the original activated carbon. The catechol adsorption isotherm at pH 7 in the activated carbon reduced (CAR) presents the formation of the monolayer to high equilibrium concentrations.

### 2.2. Species of Catechol in Solution

**Figure 1:**
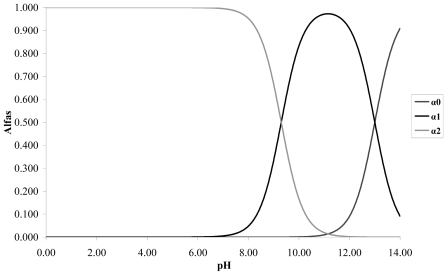
Catechol Speciation Diagram.

The respective calibration curves are determined at a wave length of 275 nm at the pH values proposed. The catechol is a weak acid with p*K*_a1_ = 9.3 and p*K*_a2_ = 13. Taking into account the distribution graph of species in Figure 1, it is observed that at pH less than 8 the predominant species is the catechol without dissociating; at pH 9 it predominates the catechol without dissociating and the anionic species *o*-hydroxy phenolate monobasic; and at pH 11 it predominates the species anionic o-hydroxy phenolate monobasic over the other two species.

### 2.3. Effect of Solution pH on Catechol Adsorption

In the following figures that present the experimental data of adsorption capacity and immersion enthalpies in function of other parameters, the lines are drawn as a guide.

**Figure 2:**
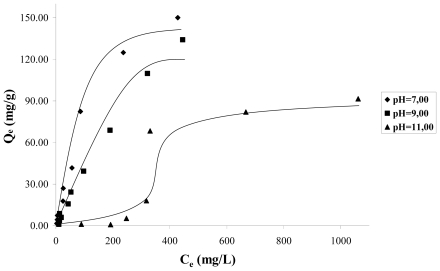
Catechol adsorption isotherms on the granular activated carbon (CAG) sample in function the pH solutions.

Figure 2, shows the adsorption isotherms at a temperature of 298 K, for solution pH values 7, 9, 11. In this figure it can be observed that as long as the solution increases the pH from 7 to 11, the mass of catechol adsorbed decreases considerably. This indicates that the adsorption is favored by the presence of the protonated species of catechol. The isotherms data in Figure 2 do not follow the behavior of the Langmuir model because the mass of cathecol adsorbed, in a wide range of concentrations as considered in this work 20–200 mg·L^−1^, is not asymptotic to high concentrations, but it continues increasing when the concentration increases. For this reason, Langmuir isotherm does not interpret correctly the isotherm data.

Based on the above, it is possible to suppose that the catechol adsorption does not occur by means of a unique mechanism and that it is a function of several types of interactions between the adsorbate and the carbon surface, thus as solution pH. At pH < 9.8, which corresponds to the pH_PZC_ for the sample CAG (see Table 2), the carbon surface is loaded positively, because the species adsorbing at pH 7 is the catechol without dissociation, which is a neutral molecule, and in these conditions the dispersive interactions determine the adsorption process.

It is observed that at pH 9, the adsorption isotherm is almost linear in the whole of the concentration interval; the adsorbed species is the catechol without dissociation and the ion o-hydroxy phenolate monobasic; and due to the positive load of the carbon surface, it intervenes in the electrostatic attraction forces between the surface and the anion that favors its accumulation.

**Figure 3:**
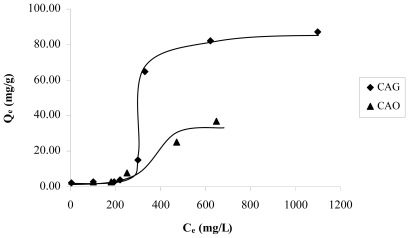
Catechol adsorption isotherms on the samples CAG and CAO at pH 11.

At pH 11, the surface is loaded negatively pH > pH_PZC_ and the found species is the anion o-hydroxy phenolate monobasic, therefore, there is repulsion between the anion and the surface. The adsorption obtained at high equilibrium concentrations of catechol, is of about 60 mg/g, when, due to the effect of a major number of particles, the electrostatic repulsive forces diminish, and possibly due to this, the adsorption to low concentrations is less than for the high concentrations, such as is observed in the adsorption isotherm. Figure 3 presents the catechol adsorption isotherms for the granular activated carbon (CAG) and, for the activated carbon oxidized CAO to pH 11, it is observed that increased content in the surface of the catechol adsorption capacity in the oxygen groups diminishes. Also, it is again observed that, with low concentrations of the solution, adsorption is minimal.

### 2.4. Effect of Chemical Surface on Catechol Adsorption

**Figure 4:**
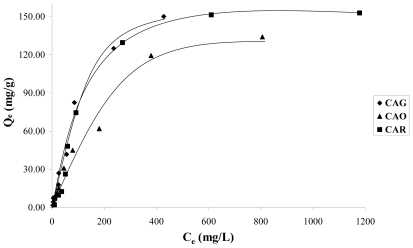
Catechol adsorption isotherms on the samples CAG, CAR and CAO at pH 7.

In order to evaluate the reduction and oxidation of the activated carbon surface in the adsorption of catechol, the isotherms of samples CAG, CAR and CAO are shown in Figure 4. It is estimated that the modification slightly increases the adsorption capacity in CAR and decreases in CAO.

The reductive treatment decreases the total acidity 1.3 times with respect to CAG. Therefore, it is expected that the concentration of π-electrons increases in the graphene layers; which means that there is an attraction between the π-electrons of the activated carbon basal planes and the aromatic ring electronic density of catechol. Because the pH solution is less than pH_PZC_, see Table 2, only dispersive forces are acting in this interaction.

The oxidation remarkably increases the quantity of acid groups that removed the electronic density of the graphene layers, this decreases the amount of adsorbed solute. This way, a great number of superficial oxygen groups increase water affinity, which would explain the low amount of retained solute. Because the solution pH is greater than 4.3 pH_PZC_, only dispersive forces are taking place in the interaction, while the repulsive electrostatic interactions between the negative charge of the activated carbon surface and the strong electronic density in π system in the aromatic ring [7,8].

The isotherms in Figure 4, in the modified activated carbons have a behavior asymptotic at high concentrations and adjust the Langmuir model better.

### 2.5. Adsorption Isotherms

The experimental data of adsorption isotherms are adjusted with Freundlich and Langmuir models. Langmuir isotherm:

$$\frac{C_{e}}{Q_{e}}=\frac{1}{{KQ}_{\text{max}}}+\frac{1}{Q_{\text{max}}}C_{e}$$

parameters: *Q_e_*—uptake equilibrium (mg·g^−1^); *K*—Langmuir constant (L·mg^−1^); *Q*_max_—monolayer adsorption capacity (mg·g^−1^); *C_e_*—solution concentration at equilibrium (mg·g^−1^).

Freundlich isotherm:

$${\mathrm{LnQ}}_{e}={\mathrm{Lnk}}_{f}+\frac{1}{n}{\mathrm{LnC}}_{e}$$

parameters: *k_f_*—Freundlich constant (mg^1−1/n^·L^1/n^·g^−1^); *n*—Freundlich exponent.

Deviation percentage is calculated as follows:

$$\%\mathrm{Desv}=\frac{1}{N}\Sigma \left|\frac{q_{cal}-q_{\text{exp}}}{q_{\text{exp}}}\right|x100\%$$

where *N* = number of experimental data [7].

In Table 3 the resultant parameters of the linearization of data adsorption are summarized by applying the models, it is important to clarify that the isotherms data that did not adjust to the models does not appear in this table. As a general trend, if *k_f_* of Freundlich model is analyzed, the value is related to the adsorption capacity and it is observed that this is greater for the solution at pH 7 in the CAG, decreasing in the following order: pH 7 > pH 9. Thus for example, *k_f_* value is 1.49 and 0.49 mg^1−1/n^·L^1/n^·g^−1^ for the catechol at pH 7 and 9, respectively. Nevertheless it is necessary to take into account the percentage of the model deviation.

The value of 1/*n* is a measurement of the surface heterogeneity. A value near 0 indicates a heterogeneous surface [17]. When the value of 1/*n* is less than 1, the adsorption process is favorable.

It is found that for the solution at pH 7, this value is less than for the solution at pH 9, but by observing the model percentage deviation, it indicates that it fits better at pH 9; therefore, the adsorption process is more favorable at pH 7.

As has already been mentioned, the catechol adsorption isotherms in function of the pH do not exhibit Langmuir model behavior since they are not prepared with asymptotic to high equilibrium concentrations (see Figure 2).

For the modifications performed in the activated carbon, it is observed that these affect the parameter values of both models, so *Q*_max_ value is greater for the CAG than for the CAR and the CAO, 238.10; 181.82 and 178.57 mg·g^−1^, respectively. Nevertheless, it is necessary to take into account that the deviation percentage of the model for this carbonaceous sample is greater than for the other two materials. Moreover it is important to mention that this value does not concur with the results showed in the adsorption isotherms (see Figure 4). Hence, it is possible to establish that the catechol adsorption order is CAR > CAG > CAO.

### 2.6. Immersion Calorimetry

The immersion enthalpy for the sample CAG in water at pH 7 was of 16.6 ± 0.8 J·g^−1^; nevertheless, for the evaluation of the produced interactions the effect of the solvent was not discounted since it is necessary to bear in mind that the influence of the species in solution that compete for the adsorption sites depend not only on the pH, but also on the adsorbate p*K*_a_ and on the pH_pzc_ of the activated carbon, which has an interesting result on the evaluation of the total effect [15,16].

**Figure 5:**
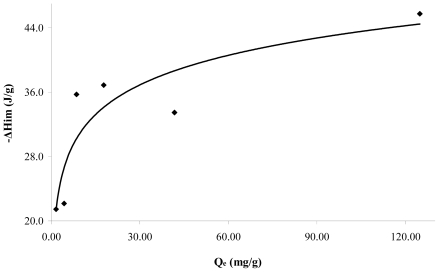
Immersion enthalpies of CAG in function of catechol adsorbed quantity at pH

Figure 5 shows the immersion enthalpies in function of the retained catechol quantity at pH 7. It is observed that the immersion enthalpy increases with the adsorbed quantity and a zone of greater interaction, absorbed at lower quantities, is obtained. Further, the immersion enthalpy is done asymptotically when the quantity retained is greater. The immersion enthalpy increases with the retained quantity from 21.5 to 45.7 J·g^−1^. This behavior is associated with the surface heterogeneity, since in principle the adsorbate occupies sites more active than those further occupied, and therefore changes the generated heat.

### 2.7. Effect of Solution pH on Immersion Enthalpy

**Figure 6:**
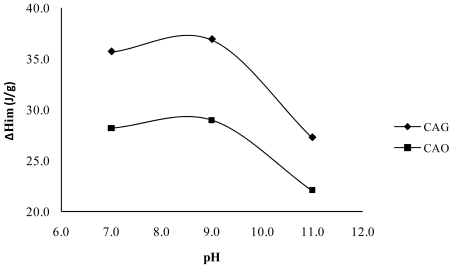
Immersion enthalpies of CAG at different pH for catechol.

Figure 6 shows the relation between the immersion enthalpies of the activated carbons CAG and CAO in a solution with an initial concentration of 100 mg·L^−1^ for the catechol and pH; values are higher for the immersion enthalpy at pH 9 than at pH 7 and the difference is due to the energetic interactions produced on the solid surface and the species present in the solution, since the not dissociated species predominate in these pH conditions. However, there is the presence of the form *o*-hidroxy phenolate monobasic, by which dispersive and electrostatic attractive interactions intervene and modify the quantity adsorbed as much as the energetic interaction in this case.

In addition, Figure 6 shows that at pH 11, lower values of immersion enthalpy are obtained. For this level of pH, the anionic species predominate and the conditions are less suitable for the adsorption, probably because the surface of the activated carbon is loaded negatively (pH > pH_PZC_) and repulsive electrostatic interactions occur.

The activated carbon CAO, presented lower adsorption values in three pH values of the experiments, indicating that the adsorption is disadvantaged with the increase of oxygenated groups on the surface of the solid and that the electrostatic repulsives forces effects a strong influence between the adsorbate and the solid.

### 2.8. Effect of Chemical Surface on Immersion Enthalpy

The immersion enthalpies were also determined with the activated carbons modified by means of reductive and oxidative treatment at pH 7 for an initial concentration of 1500 mg·L^−1^, that corresponds to the maximum concentration used in this work, and which have the highest values of immersion enthalpy.

**Figure 7:**
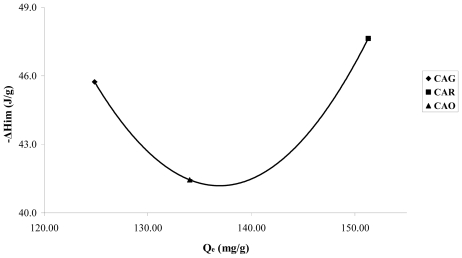
Immersion enthalpies of carbons in function of catechol adsorbed quantity

A small increase of the energetic interactions in the CAR produced between the catechol molecule and the carbon surface can be observed in Figure 7; an effect attributed basically to the surface chemistry and demonstrates the nature of the possible adsorption sites.

Sample CAR shows a small increase in the immersion enthalpies; the catechol is in its molecular form and intervenes mainly in dispersive interactions in the adsorption process. As this sample has basic character pH_PZC_ 10.1 and low concentration of functional oxygenated acid groups, the energetic interactions take place basically with the electrons π of the graphene layers.

In sample CAO, Figure 7a small decrease in the immersion enthalpy for CAO is observed in comparison to CAG, with values of 45.7 ± 2.3; 47.6 ± 2.4 and 41.4 ± 2.1 J·g^−1^ for CAG, CAR and CAO, respectively. The results obtained indicate that the enthalpic interactions are similar for the three activated carbon to high concentration; nevertheless it is interesting to estimate that the trends complement the information obtained in the isotherms.

## 3. Experimental Section

A granular activated carbon Carbochem^TM^—PS230 is used for this experiment. The precursor of this carbon is coconut shell (sample CAG).

### 3.1. Modification of the Granulated Activated Carbon

*Hydrogen atmosphere heat treatment* (sample CAR): About 20 g of granulated activated carbon are placed on the reducing system with hydrogen. The temperature is increased to 393 K; the set becomes vacuum about 10^−4^ mmHg. Next the hydrogen is introduced. In these conditions, the oven is warmed progressively to 573 K for 6 days. Finally the activated carbon is stored in a nitrogen atmosphere.

*Treatment with nitric acid* (sample CAO): Six grams of the granulated activated carbon are placed with a solution of nitric acid of 7 mol·L^−1^ in soxhlet equipment for nine hours at the temperature of the solution boiling, using 60 mL of acid per gram of activated carbon. The sample is then washed with distilled water until a constant pH value is obtained and next the activated carbon is dried at 383 K for 24 h. Finally the samples are stored in a closed container in nitrogen atmosphere.

### 3.2. Textural and Chemical Analysis of the Original and Modified Activated Carbons

The carbonaceous samples (measuring about 0.100 g) are degasified at 523 K for a period of 3 h, to clean the surface before the nitrogen adsorption, in an Autosorb 3B, Quantachrome Co. The corresponding adsorption nitrogen isotherms are obtained with this equipment at 77 K. In addition, the acid and basic sites are determined by Boehm [15] acid-base titration method and the point of zero charge is determined by the mass titration method [18].

### 3.3. Adsorption in the Aqueous Phase

Catechol concentration in aqueous solution is determined by a UV spectrophotometric method. The maximum absorbance wavelength is determined at pH values 7, 9, 11 in a Thermospectronic Genesys 10. The adsorption isotherm data are obtained by putting 0.500–0.250 g of the carbonaceous samples in contact with a 50 mL volume of catechol solutions at known initial concentrations ranging from 20 to 2000 mg·L^−1^, with pH adjustment at 298 K for 48 h.

### 3.4. Immersion Calorimetry

#### 3.4.1. Determining Immersion Enthalpy

Catechol solutions: 50.0 mL of aqueous solutions of catechol at pH 7, at concentrations ranging from 20 to 1500 mg·L^−1^ are placed in the calorimetric cell and between 0.500–0.250 g of the carbonaceous samples are weighed in a glass cell; the cell is assembled and the setting allows it to equilibrate for approximately 40 min. When the variation of the exit electrical resistance thermistor is constant, the readings start at an initial period of 15 min, with resistance readings every 20 s; soon the carbonaceous samples are put in contact with the catechol solutions, the resistance readings are continued until they remain constant and, finally, they are electrically calibrated [12].

Water: The immersion enthalpy measurements of the carbonaceous material are taken in distilled water at pH 7, in the same way that the earlier procedure.

## 4. Conclusions

The results show that the oxidation and reduction treatments carried out, do not demonstrate valuable changes in the textural characteristics of the carbonaceous materials, but they do so in the surface electrochemical properties.

The catechol adsorption capacity depends on the pH solution; the adsorption isotherm decreases when the pH increases from 7 to 11 and the maximum adsorption at pH 7 in the CAG is obtained. The chemical oxidation and reduction modifications to the activated carbon surface slightly increase the adsorption capacity in CAR and decrease in CAO.

The values of the immersion enthalpies increase with the quantity adsorbed; for example, the immersion enthalpy for the catechol increases with the quantity retained from 21.5 to 45.7 J·g^−1^.

The results show a variation in immersion enthalpy, based on the adsorbed quantity and on the initial concentration of the solution, similar to what occurs in the adsorption isotherms. It can therefore be concluded that the intensity of the interaction changes in function of the liquid phase composition.

## Figures and Tables

**Table 1:** Activated Carbons Textural Characteristics.

Sample	BET Surface Area (m^2^·g^−1^)	Micropore volume (cm^−1^)	Mesopore volume (cm^3^·g^−1^)
CAG	1140	0.51	0.12
CAR	1171	0.56	0.12
CAO	1181	0.56	0.09

**Table 2:** Superficial chemistry of the activated carbons.

Sample	pH_PZC_	Total Acidity (meq·g^−1^)	Total Basicity (meq·g^−1^)
CAG	9.8	0.30	0.60
CAR	10.1	0.20	0.61
CAO	4.3	1.26	0.25

**Table 3:** Parameter values of the Langmuir and Freundlich models for the catechol adsorption on the CAG and carbons modified at pH 7.

		Langmuir				Freundlich			
		
Adsorbate	pH	*Q*_max_ (mg·g^−1^)	*K* (L·mg^−1^)	*R*^2^	%Des	*k_f_* (mg^1−1/n^·L^1/n^·g^−1^)	1/*n*	*R*^2^	%Desv
	7	238,10	4, −3	0.90	0.21	1.49	0.81	0.96	3.15
CAG	9	--	--	--	--	0.49	0.94	0.97	2.47
	11	--	--	--	--	--	--	--	--
CAR	7	181.82	5.7E–3	0.97	3.61	1.39	0.74	0.87	8.58
CAO	7	178.57	3.9E–3	0.97	0.09	1.62	0.70	0.96	1.79
	11	--	--	--	--	0.002	1.50	0.92	6.23
